# Cross-cultural adaptation and search for evidence of validity of the brazilian version of the nonreligious-nonspiritual scale (NRNSS)

**DOI:** 10.1186/s41155-025-00350-5

**Published:** 2025-06-13

**Authors:** André Gadelha-Weyne, Ícaro Moreira Costa, Daniel Foschetti Gontijo, Tauily Claussen D´Escragnolle Taunay, Ryan Cragun

**Affiliations:** 1 Universidade de São Paulo, Av. Professor Mello Moraes, 1721, CEP 05508030 São Paulo/SP, Brasil; 2 Universidade de Fortaleza, Av. Washington SoaresEdson Queiroz, 1321, CEP 60811905 Fortaleza, CE Brasil; 3 Instituto Ponto Azul, Av. Raja Gabáglia, 1613 (501), Belo Horizonte/MG, CEP 30380435 Brasil; 4 Universidade de Fortaleza, Av. Washington Soares, 1321, Fortaleza/CE, 60811905 Brasil; 5 University of Tampa, 401, W. Kennedy Blvd, Tampa, FL Box 147 F., USA

**Keywords:** Atheism, Cross-cultural adaptation, Psychometrics, Religion, Religiosity, Scale validation, Secularism, Spirituality

## Abstract

**Background:**

The number of non-religious people in the world has increased, which justifies the development and validation of good instruments to assess secularism, i.e., the absence of religiosity and spirituality.

**Objective:**

The present study aimed to develop a cross-cultural adaptation and search for evidence of the validity of the nonreligious-nonspiritual scale (NRNSS).

**Methods:**

For a congruent scale translation, a cross-cultural adaptation was performed. The search for evidence of validity was carried out through the following steps: (1) evidence of validity based on the internal structure, where an exploratory factor analysis (EFA) and confirmatory factor analyses (CFA) were performed, and the internal consistency was verified of the factors obtained; (2) evidence of validity based on converging relationships with external measurements, where correlations were made between the NRNSS factors and the Brazilian Portuguese version of the Duke Religiosity Index (P-DUREL) and the World Health Organization’s Quality of Life Instrument-Spirituality, Religion and Personal Beliefs module (WHOQOL-SRPB); and (3) comparison of self-identification categories with NRNSS outcomes using two one-way ANOVA tests, comparing scale scores between different groups (e.g., atheists, agnostics, spiritualists, and religious people). The data collection process took place through a link shared on social networks allowing access to the structured questionnaire.

**Results:**

The present study obtained the following results: (1) the EFA supported a one-factor model for the scale, but (2) the CFA presented satisfactory indices for the the model composed of two factors, non-religiosity (NR) and non-spirituality (NS); (3) internal consistency indices greater than 0.95 were obtained in all factors indicated in both tested models; (4) all analyzed correlations obtained results as expected, indicating that the scale actually measures the proposed constructs; and (5) decreasing levels of NR and NS were obtained according to the respective beliefs: atheists, agnostics, spiritualists, and religious people.

**Conclusion:**

The NRNSS presented favorable psychometric properties, enabling it to be used with two different factors (NR and NS).

## Introduction

Contemporary studies of religion show a lack of conceptual consensus in the field regarding the measurement of religiosity and spirituality (Freitas, 2017; Koenig, 2008). In recent years, new definitions of these terms have emerged. As argued by Zinnbauer and Pargament (2005), these definitions have been shaped by the decline of traditional religious institutions and the increase in individualized manifestations of faith. This development proved to be fertile enough to lead to a distinction between two dimensions in the search for contact with the sacred: religiosity and spirituality. Religiosity is increasingly understood as a personal or group search for the sacred that takes place in a traditional, organized religious context. Spirituality is increasingly understood as a personal or group search for the sacred outside the context of traditional, organized religion (Zinnbauer & Pargament, 2005).

### The study of nonreligious individuals

In most of the Western World, there is a growing number of people who consider themselves nonreligious (Smith & Cragun, 2019). Nonreligion can be considered as any position, perspective, or practice that is primarily defined by (or in relation to) religion but which is nevertheless considered to be other than religion (Lee, 2012).

The growth of the nonreligious can be observed in international surveys. Keysar (2017), using the World Values Survey, found that 53% of adults claimed to be religious, while 33% claimed to be nonreligious and 11% identified as atheists. Likewise, Bullivant (2018), in a survey conducted in 22 European countries, found that nonreligious are numerically higher than religious in more than half of the countries surveyed. Cragun et al. (2018) observed generational differences in religiosity, with students’ levels being 12% lower than their fathers’ and 17% lower than their mothers’. In Brazil, the number of nonreligious individuals grew from 0.8% in 1970 to 8.3% in 2010 (Mariano, 2013). As these studies illustrate, the nonreligious are growing in much of the world, illustrating the importance of developing new ways to measure how nonreligious and nonspiritual individuals are.

However, the non-religious group has multifaceted experiences regarding belief and disbelief in God, especially in increasingly secularized societies (Lindeman et al., 2020). For this reason, the distinction between religiosity and spirituality becomes strongly well suited to the empirical evidence obtained by the researchers. The foundational study by Cragun et al. (2015), which developed the nonreligious-nonspiritual scale (NRNSS), demonstrated that religiosity and spirituality could be measured independently, as well as the study in a Greek sample (Polemikou et al., 2019). This happens because some nonreligious may hold spiritual/paranormal/supernatural beliefs, for this reason being called as spiritual-but-not-religious (SBNR). For example, in a study with Americans, SBNRs scored higher on supernatural beliefs and mystical experiences than the broad group of nonreligious and similar to the religious, but with greater magical ideation (Willard & Norenzayan, 2017). Bullivant et al. (2019) also found that, in a sample of Brazilians, 11% of non-believers classified themselves as “spiritual but not religious,” highlighting the complex network of beliefs and self-identification of this group, since those who do not hold a belief in God can also consider themselves as spiritual but not religious, but still reinforcing the need to consider spirituality and religiosity as two independent and interconnected constructs.

### Measurement limitations

In light of the growth of the nonreligious, there has been a growing interest in studying nonreligious people (Coleman III & Jong, 2021). Some authors (Coleman III et al., 2015; Smith & Cragun, 2019; Cragun, 2019) have argued that the psychology of religion is incomplete without proper consideration of individuals who identify as nonreligious, highlighting the need for instruments that can validly assess non-religiosity and non-spirituality. Developing such tools is essential for advancing research that addresses gaps in understanding the impacts of religious diversity on social behavior. Moreover, these instruments play a crucial role in promoting social understanding by accurately representing nonreligious populations, thereby informing public policies and interventions that respect and address their specific needs. Nonetheless, the creation of these instruments has encountered several challenges.

Among the difficulties encountered, the formulation of unsatisfactory items in instruments has been highlighted (Cragun et al., 2015; Meezenbroek et al., 2012; Zwingmann et al., 2011). Meezenbroek et al. (2012) draw attention to the fact that spirituality is regularly included in questionnaires with overly vague terms. In addition, some instruments have presented items with ambiguous meaning and implicitly assume that the respondent is religious and/or has spiritual practices/beliefs (Cragun, 2019; Cragun et al., 2015). Given these methodological limitations, Cragun and colleagues (2015) pointed to the need for a new instrument that can validly and reliably assess religiosity and spirituality in religious and non-religious people. They also highlighted that there is a lack of instruments capable of identifying the emerging category of spiritual but not religious (“SBNR” but also called “spiritualists”). Prior research has suggested that the only way to find this information has been through questions of self-identification (Koenig, 2008; Vitorino et al., 2018), which has been pointed argued problematic as an ad hoc measure, particularly when there are better measurement instruments (Cragun, 2019).

In a recent systematic review, Forti et al. (2020) examined the instruments used to measure religiosity/spirituality (R/S) in Brazil in the context of health. They examined 16 R/S measurement instruments available in Brazil (i.e., in Brazilian Portuguese), which contained some spirituality measures, but none with the specific aim of measuring religiosity and spirituality as distinct constructs.

### The nonreligious-nonspiritual scale

In order to fill the aforementioned gap in the literature, Cragun and colleagues (2015) developed the nonreligious-nonspiritual scale (NRNSS), an instrument whose 17 items were originally clustered into two factors: institutional religiosity and individual spirituality. The scale is capable of measuring (non)religion and (non)spirituality separately, on a spectrum between their presence and absence. As a result, the scale can clearly distinguish between four possible groups: religious and spiritual (RS), spiritual but not religious (SBNR), religious but not spiritual (RNS), and nonreligious and nonspiritual (NRNS). The NRNSS measures religiosity and spirituality separately in the same questionnaire. Using the same definitions of religiosity and spirituality adopted by the present study, the authors of the scale indicated that the non-religiosity (NR) and non-spirituality (NS) poles of the continuum refer to what is different or the opposite of what is religious or spiritual (Cragun et al., 2015; Polemikou et al., 2019). Therefore, although Brazil is a mostly religious country, it is hoped that the adapted scale can contribute to Brazilian studies on the (non)religious population, given its theoretical structure of measuring spirituality separately from religiosity. In this sense, the present research aimed to carry out a cross-cultural adaptation of the instrument and search for evidence of validity of the nonreligious-nonspiritual scale (NRNSS) for the Brazilian population.

## Methods

### Translation and cross-cultural adaptation

The process of translating and adapting the scale was based on the methodological guidelines proposed by Beaton (2000), Borsa et al. (2012), and Borsa and Seize (2017) in six distinct stages, namely (1) translation, (2) synthesis, (3) committee of experts, (4) target audience assessment, (5) reverse translation, and (6) presentation of documents to authors of the original instrument.

In the first stage, the original scale was translated by two Brazilian translators fluent in the source language (English). Independently, they translated the texts and items into Portuguese. In the second stage, a virtual meeting was held between the two translators and the researchers, at which point the translators reached a consensus on a synthesis of the Portuguese version. In the third stage, the translated and synthesized version of the scale was submitted to a committee of judges, composed of three academic researchers in the psychology of religion. In the fourth stage, an assessment of the target audience was carried out using 19 people with a (non)religious profile and diverse sociodemographic characteristics. At this stage of the research, the scale was presented using a provisional version following an interview, in which the participants were asked about the clarity and limitations of the instrument. In the fifth stage, a reverse translation of the final version of the NRNSS was carried out, repeating the method of independent translation and consensual synthesis. Finally, the sixth stage consisted of presenting the questionnaire to the original author, who approved without reservation the Brazilian version of the NRNSS. It should be noted that, in addition to translating the items of the instrument, the preliminary explanation of what spirituality means was also translated, as presented in the original scale. This ensured that participants understood the concept of spirituality before responding to the scale.

### Participants

To validate the Brazilian version of the NRNSS, we gathered a non-probabilistic convenience sample of 960 respondents. Inclusion criteria were being over 18 years of age, being literate, and having agreed to the free and informed consent terms.

Most of the 960 participants were women (*n* = 495; 51.6%), and their ages ranged from 18 to 86 (*M* = 37.65; SD = 14.57), with a high proportion of single individuals (*n* = 471; 49.1%). Many of them resided in the Southeast region of Brazil (*n* = 446; 46.5%) and had completed a postgraduate degree (*n* = 285; 29.7%). Family income was between five and ten times the minimum wage (*n* = 320; 33.3%), and, regarding religious affiliation, there was a predominance of those who do not follow any religion (*n* = 617; 64.3%) followed by Catholics (*n* = 105; 10.9%) (see Table 1).

**Table 1:** Sample characteristics

Variable		n	%
Region	North	26	2,7
	Northeast	293	30,5
	Midwest	59	6,1
	Southeast	446	46,5
	South	136	14,2
Gender	Male	458	47,7
	Female	495	51,6
	Non-binary	7	0,7
Marital Status	Single	471	49,1
	Married/Civil Partnership	408	42,5
	Divorced	69	7,2
	Widowed	12	1,3
Education	Complete or incomplete primary education	13	1,3
	Complete or incomplete secondary education	117	12,2
	Incomplete higher education	217	22,6
	Complete higher education	254	26,5
	Incomplete post-graduate studies	74	7,7
	Complete post-graduate studies	285	29,7
Income	Less than $530 USD (Less than 2 minimum wages in Brazil)	154	16,0
	$795 to $1,055 USD (3 to 4 minimum wages in Brazil)	239	24,9
	$1,320 to $2,640 USD (5 to 10 minimum wages in Brazil)	320	33,3
	$2,905 a $5,280 USD (11 to 20 minimum wages in Brazil)	134	14,0
	Above $5,280 USD (Above 20 minimum wages in Brazil)	51	5,3
	Prefer not to disclose	62	6,5
Belief	I am an atheist: I believe that God and/or any spiritual dimension does NOT exist	299	31,1
	I am agnostic: I do not believe in the existence of God and/or the spiritual dimension, but I also do not disbelieve	183	19,1
	I am spiritual but not religious: I believe in the existence of God and/or in a spiritual dimension, but I do not follow any religion	163	17,0
	I am religious: I believe in the existence of God and/or in a spiritual dimension and I follow a religion	279	29,1
Religion	Catholic	105	10,9
	Evangelical/Protestant	71	7,4
	Spiritist/Kardecist	101	10,5
	Afro-descendant religions (Candomblé/Umbanda/etc.)	23	2,4
	No religion	617	64,3
	Others	43	4,5

### Instruments

#### Nonreligious-nonspiritual scale (NRNSS)

This was developed by Cragun et al. (2015) and aims to separately measure non-religiosity and non-spirituality in individuals, enabling the identification of different belief profiles, such as religious, spiritual, non-religious, and non-spiritual. The scale comprises 17 items, divided into two factors: non-religiosity (items 1 to 8) and non-spirituality (items 9 to 17).

The NRNSS is answered using a 5-point Likert scale, ranging from “strongly disagree” to “strongly agree.” For the assessment, the average score for each factor is calculated, also ranging from 1 to 5 points, allowing for a detailed analysis of the dimensions of religiosity and spirituality independently. This structure provides data for studies that explore the diversity of beliefs and their impacts in different contexts.

#### Religious/spiritual identity

To identify the participants’ belief/disbelief and identity regarding the existence of God and/or a spiritual dimension, we created an item with 5 response options. These options were “I am an *atheist*: I believe that God and/or any spiritual dimension does NOT exist”, “I am an *agnostic*: I do not believe in the existence of God and/or the spiritual dimension, but I also do not disbelieve,” “I am a *spiritualist*: I believe in the existence of God and/or in a spiritual dimension, but I do not follow any religion,” “I am *religious*: I believe in the existence of God and/or in a spiritual dimension and I follow a religion” and “Neither of these is my case.” The largest portion of our sample was atheists (*n* = 299; 31.1%), followed by religious individuals (*n* = 279; 29.1%), agnostics (*n* = 183; 19.1%), spiritualists (*n* = 163; 17%), and “others” (*n* = 37; 3.7%). This last group was excluded from the analyses.

#### The Duke Religiosity Index (DUREL)

This instrument contains five self-report items that seek to measure religiosity using factors of organizational religiosity (OR), non-organizational religiosity (NOR), and intrinsic religiosity (IR) (Taunay et al., 2012).

### The WHOQOL-SRPB

It was used only for the SRPB session. This is a questionnaire developed by a team from the World Health Organization (WHO) that sought to assess how spirituality, religiosity, and personal beliefs (SRPB) relate to quality of life (Fleck & Skevington, 2007). The instrument contains 38 self-report items, arranged into 8 factors: (1) spiritual connection, (2) faith, (3) spiritual strength, (4) inner peace, (5) totality and integration, (6) meaning in life, (7) hope and optimism, and (8) admiration (Panzini et al., 2011).

### Data collection

With approval from the Research Ethics Committee at the University of Fortaleza (CAAE: 50,651,721.0.0000.5052), data collection took place entirely through an online platform. The questionnaire link was published on various social networks (e.g., Facebook, Twitter, etc.). The questionnaire was hosted on the Google Forms platform. Data collection took place during the month of October 2021.

### Data analysis

The analyses are divided into five stages. The first and second stages consisted of the search for evidence based on the internal structure of the NRNSS, the third stage examined the normality of the instrument, and the fourth and fifth stages concerned the search for evidence-based on relationships with external measurements. In the first stage (1), two exploratory factor analyses (EFA) were carried out using JASP. We aimed to verify the factorial structure of the scale. For the first EFA, a polychoric matrix and the robust diagonally weighted least squares (RDWLS) extraction method were used (Asparouhov & Mthen, 2010). Regarding the choice of factor retention, the criteria of the parallel analysis with a random permutation of the collected statistics were used (Timmerman & Lorenzo-Seva, 2011) and the rotation used was Oblimin Promax. The reliability of the factors was checked with McDonald’s Omega.

In the second stage (2), a confirmatory factor analysis (CFA) was carried out, using JASP software, in order to verify the plausibility of the multifactorial structure of the NRNSS. The procedure was configured using the robust diagonally weighted least squares (RDWLS) estimation method, suitable for categorical data (DiStefano & Morgan, 2014; Li, 2016). The adjustment indices used were *X*^2^, *X*^2^/dl, Comparative Fit Index (CFI), Tucker-Lewis Index (TLI), standardized root mean residual (SRMR), and root mean square error of approximation (RMSEA). Values of *X*^2^ should not be significant; the *X*^2^/df ratio should be < 5 or, preferably, < 3; CFI and TLI values must be > 0.90 and preferably above 0.95; RMSEA values should be < 0.08 or, preferably < 0.06, with a confidence interval (upper limit) < 0.10 (Brown, 2015); and; SRMR should be close to 0 to indicate a good fit (Cho et al., 2020). Reliability was measured using McDonald’s omega. It is noteworthy that, prior to the factor analyses, we split the sample using the cross-validation technique, resulting in a sample of 480 participants for each step.

In the third stage (3), descriptive statistical analyses of the sociodemographic data were performed, at which time the normality of the results referring to the NRNSS factors was verified through the Kolmogorov–Smirnov (KS) and Shapiro–Wilk (SW) tests. In the fourth stage (4), the search for evidence of validity was undertaken based on relationships with external measurements. This analysis included two convergent measurement ratios. The first analyzed the correlation between the results of the NRNSS and DUREL, and the second through the correlation between the NRNSS and the SRPB. In both cases, Spearman’s correlation was used, in light of the non-parametric conditions of the data.

Finally, in the last step (5), two one-way ANOVA tests were performed in order to assess whether there were differences in the levels of non-religiosity and non-spirituality between people of different religious/spiritual identities (i.e., atheists, agnostics, spiritual but not religious and religious). We expected atheists to present the highest level of non-spirituality and non-religiosity, followed by agnostics, spiritualists, and religious people. The third, fourth, and fifth steps were performed using IBM SPSS version 23.

## Results

### NRNSS validity evidence based on internal structure

Our first EFA indicated the NRNSS presented a satisfactory confidence index (KMO = 0.977) and Bartlett’s sphericity test (23,086.147, gl = 136,000, *p* < 0.001). Through parallel analysis and Oblimin Promax rotation, a one-factor model was obtained, accounting for 74.6% of the total variance of the construct. Favorable internal consistency indexes of McDonald’s omega were found (*ω* = 0.98), as well as clustering of all 17 items of the instrument with satisfactory factor loadings (all greater than 0.40; see Table 2).

**Table 2:** Factorial structure of the Nonreligious-Nonspiritual Scale (NRNSS)

Item	Factor loading	Communalities
1. Eu sou guiado pela religião ao tomar decisões importantes em minha vida.	0.863	0.747
2. A religião é a melhor referência do que é certo e errado.	0.782	0.612
3. Quando enfrento dificuldades em minha vida, busco apoio na religião.	0.911	0.831
4. Eu me engajo em práticas religiosas.	0.883	0.783
5. A religião me ajuda a responder muitas perguntas que tenho sobre o sentido da vida.	0.916	0.839
6. Eu me descreveria como uma pessoa religiosa.	0.897	0.804
7. A religião é necessária para minha felicidade pessoal.	0.890	0.792
8. Eu me incomodaria se um filho meu quisesse se casar com uma pessoa que NÃO é religiosa.	0.608	0.369
9. A espiritualidade é importante para mim.	0.891	0.794
10. O que eu faço de certo ou errado vai afetar o que acontece comigo na vida após a morte.	0.859	0.738
11. Eu tenho um espírito/essência para além do meu corpo físico.	0.872	0.761
12. O universo tem uma origem espiritual.	0.917	0.841
13. Existem benefícios que apenas a espiritualidade pode proporcionar.	0.795	0.632
14. A dimensão espiritual existe.	0.887	0.787
15. Eu me engajo em atividades espirituais.	0.886	0.785
16. Eu sinto uma sensação de conexão com algo além do que podemos observar, medir ou testar cientificamente.	0.874	0.764
17. Eu só consigo encontrar um sentido válido para a vida por causa da espiritualidade.	0.883	0.780
McDonald's Omega	0.98	

Our next step was to conduct the CFA. Both the model proposed by the EFA (model 1) and a new model (model 2) were tested. The latter was consistent with that proposed by the theory that underlies the conceptual distinction between religiosity and spirituality (Lee, 2012; Zinnbauer & Pargament, 2005), adopted in the original instrument as two distinct factors (Cragun et al., 2015). The CFA results showed that the proposed single-factor structure fit the data well. However, even though all the other fit indices supported the model, it is worth noting that a significant chi-square value and an index of 2/df above 0.3 were obtained, contrary to the recommendations of the literature (Brown, 2015).

**Table 3:** Fit indices of the confirmatory factor analyses

X² (df)	X²/df	CFI	TLI	SRMR	RMSEA (90% IC)
168.370 (118) (n.s.)	1.37	1.000	1.000	0.01	0.03 (0.01–0.04)

** p < 0.05; n.s. non-significant, M Model, X^2^ chi-square; df degrees of freedom, CFI Comparative Fit Index, TLI Tucker-Lewis Index, SRMR Standardized Root Mean Square Residual, RMSEA Root Mean Square Error of Approximation

**Fig. 1:**
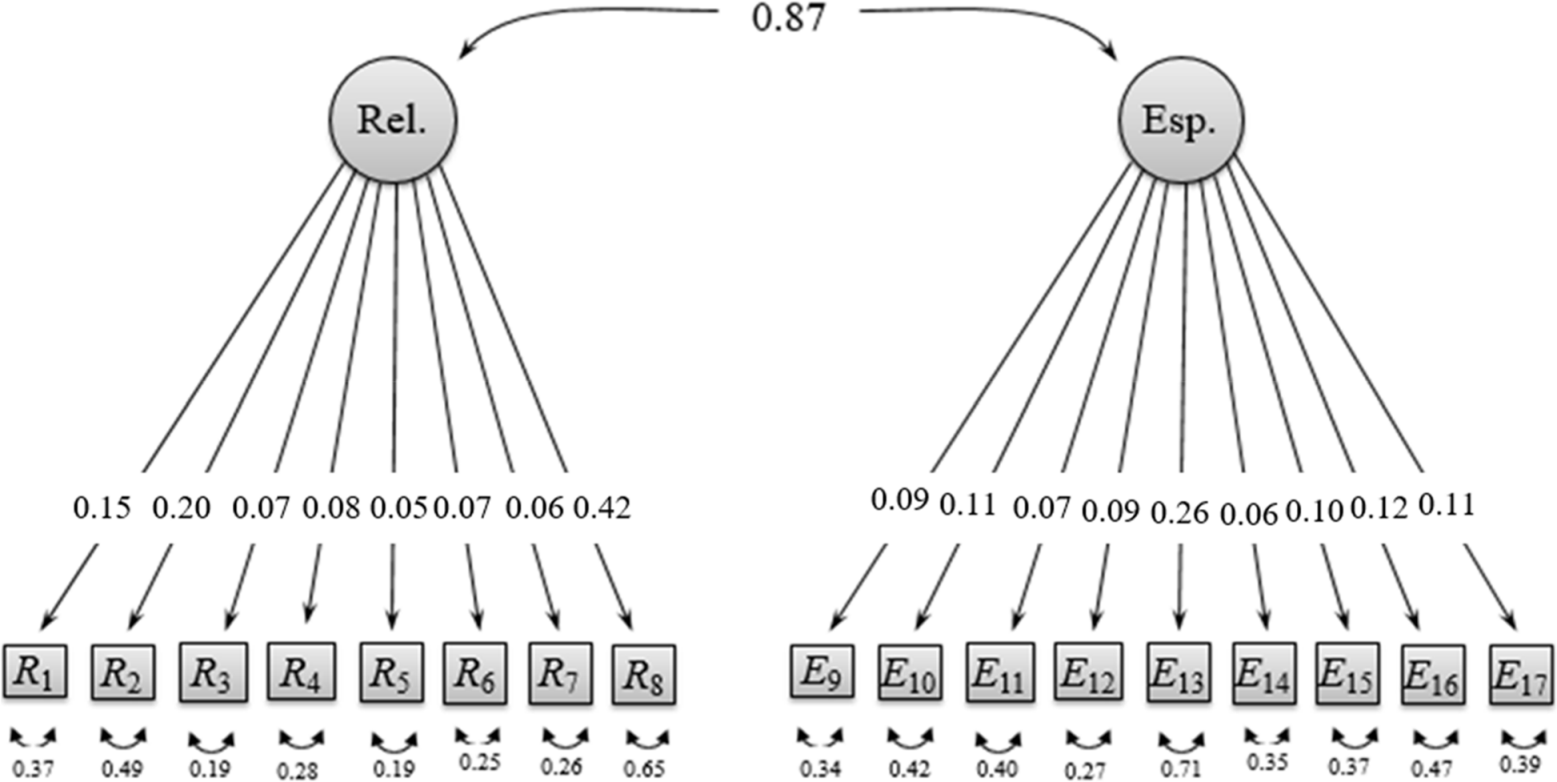
NRNSS structure and factor loadings

When analyzing the results of the two-factor structure, fit indices indicated strong support for this model, with non-significant chi-square and *χ*^2^/df less than 0.3 (see Fig. 1; see Table 3). The first factor consists of items referring to non-religiosity (items 1 to 8), whereas the second is composed of non-spirituality items (items 9 to 17). The McDonald’s omega indices obtained in the non-religiosity (ω = 0.97) and non-spirituality (ω = 0.97) factors were favorable. Additionally, the factors of non-religiosity and non-spirituality showed a very strong correlation (r = 0.87; *p* < 0.001; see Fig. 1), suggesting the close relationship between these constructs.

### NRNSS validity evidence based on convergent relationship with external measurements

In order to verify the validity of the NRNSS based on relationships with convergent external measurements, we correlated data from the NRNSS, the Duke Religiosity Index, and the SRPB factors (see Table 3). The distribution tests did not indicate normality in the non-religiosity (KS = 0.17, *p* < 0.001; SW = 0.84, *p* < 0.001) and non-spirituality (KS = 0.12, *p* < 0.001; SW = 0.90, *p* < 0.001) variables. The data presented in Table 3 indicate that all correlations between non-religiosity and non-spirituality and the three religiosity factors measured by the Duke Religiosity Index were considered strong and negative, as expected, highlighting the correlation between NRNSS factors and intrinsic religiosity. Table 3 also shows that all correlations between NRNSS and SRPB factors were significant. The following correlations between NR and NS and SRPB factors stand out: connection with a spiritual being, spiritual strength, and faith, which are those most closely related to the concept of spirituality.

Regarding the fifth stage of data analysis, two one-way ANOVA tests were performed (see Table 4). These tests verified that the levels of non-religiosity and non-spirituality corresponded with religious/spiritual groups (i.e., atheist, agnostic, spiritual but not religious, and religious).

**Table 4:** Spearman correlation analysis between NRNSS and SRPB data

	NR	NS	OR	NOR	IR	CON	ML	ADM	TOT	SSTR	INP	HO	F
NR	-												
NS	0.860*	-											
OR	− 0.787*	− 0.717*	-										
NOR	− 0.769*	− 0.776*	0.711*	-									
IR	− 0.885*	− 0.864*	0.773*	0.824*	-								
CON	− 0.838*	− 0.895*	0.715*	0.795*	0.879*	-							
ML	− 0.620*	− 0.700*	0.577*	0.624*	0.689*	− 0.708*	-						
ADM	− 0.441*	− 0.538*	0.406*	0.461*	0.504*	0.545*	0.637*	-					
TOT	− 0.590*	− 0.670*	0.532*	0.604*	0.671*	0.680*	0.731*	0.632*	-				
SSTR	− 0.802*	− 0.887*	0.680*	0.773*	0.861*	0.901*	0.739*	0.609*	0.730*	-			
INP	− 0.240*	− 0.251*	0.248*	0.301*	0.324*	0.297*	0.463*	0.429*	0.548*	0.362*	-		
HO	− 0.379*	− 0.416*	0.363*	0.407*	0.466*	0.467*	0.596*	0.522*	0.590*	0.507*	0.634*	-	
F	− 0.832*	− 0.864*	0.720*	0.797*	0.887*	0.897*	0.704*	0.532*	0.685*	0.895*	0.333*	0.513*	-

*p < 0.01; *NR* non-religiosity, *NS* non-spirituality, *OR* organizational religiosity, *NOR* non-organizational religiosity, *IR* intrinsic religiosity, *CON* connection with spiritual being, *ML* meaning in life, *ADM* admiration, *TOT* totality and integration, *SSTR* spiritual strength, *INP* inner peace, *HO* hope and optimism, *F* faith

The ANOVA results showed that there were significant differences between the groups, both in terms of non-religiosity [Welch’s* F*(4, 191.86) = 812.413, *p* < 0.001], and non-spirituality [Welch’s *F* (4, 197.990) = 1268.844, *p* < 0.001] (see Table 5. The Games-Howell post-hoc test, interpreted through bootstrapping procedures, showed that differences were found in the levels of non-religiosity and non-spirituality among all groups. Specifically, atheists presented the highest level of non-spirituality and non-religiosity, followed by agnostics, spiritualists, and religious people (Table 6).

**Table 5:** Descriptive statistics of non-religiosity and non-spirituality for the total sample and separated by groups

			Descriptive statistics	Bootstrapping estimates		
				Confidence interval (95% IC Bca)		
				Standard error	Inferior limit	Superior limit
Atheists	NR	*M*	4.89	0.01	4.87	4.92
		*SD*	0.20	0.01	0.17	0.23
	NS	*M*	4.76	0.01	4.72	4.80
		*SD*	0.35	0.02	0.31	0.38
Agnostics	NR	*M*	4.59	0.03	4.50	4.62
		*SD*	0.45	0.02	0.40	0.51
	NS	*M*	3.89	0.04	3.80	3.99
		*SD*	0.64	0.02	0.59	0.68
Spiritualists	NR	*M*	3.92	0.05	3.81	4.02
		*SD*	0.69	0.04	0.61	0.77
	NS	*M*	2.40	0.06	2.27	2.53
		*SD*	0.82	0.05	0.73	0.91
Religious	NR	*M*	2.32	0.04	1.23	2.41
		*SD*	0.75	0.02	0.69	0.79
	NS	*M*	1.80	0.03	1.72	1.88
		*SD*	0.65	0.03	0.59	0.71
Total sample	NR	*M*	3.89	0.03	3.81	3.96
		*SD*	1.20	0.02	1.15	1.24
	NS	*M*	3.27	0.04	3.18	3.37
		*SD*	1.37	0.01	1.34	1.40

*NR* non-religiosity, *NS* non-spirituality, *M* mean, *SD* standard deviation

In summary, the results from the one-way ANOVA tests corroborated the hypothesized relationship between levels of non-religiosity and non-spirituality and the categories of religious/spiritual identity. This result indicates that the NRNSS converges with the objectives proposed by Cragun et al. (2015).

## Discussion

We successfully cross-culturally adapted the NRNSS and provided evidence of its validity. While the EFA supported a one-factor model, the CFA indicated that the two-factor structure, as proposed by Cragun et al. (2015), demonstrated superior psychometric properties, particularly compared to the one-factor model. This same conclusion was also presented by Cragun et al. (2015), who indicated that the great conceptual convergence of religiosity and spirituality could lead to an overlap of these in a second-order factor.

**Table 6:** Games-Howell post-hoc test with Bootstrapping in non-religiosity and non-spirituality (95% CI Bca)

Comparisons between groups		Mean difference (NR)	Mean difference (NS)	Bootstrapping estimates (95% CI BCa)					
				Standard error (NR)	Standard error (NS)	Inferior limit (NR)	Superior limit (NR)	Inferior limit (NS)	Superior limit (NS)
Atheists	Agnostics	0.33	0.86	0.03	0.05	0.25	0.40	0.76	0.97
	Spiritualists	0.97	2.35	0.05	0.06	0.87	1.08	2.22	2.49
	Religious	2.57	2.95	0.04	0.04	2.47	2.66	2.86	3.04
Agnostics	Spiritualists	0.64	1.49	0.06	0.07	0.52	0.76	1.33	1.64
	Religious	2.23	2.09	0.05	0.06	2.13	2.34	1.97	2.20
Spiritualists	Religious	1.59	0.59	0.06	0.07	1.46	1.72	0.44	0.75
*NR* non-religiosity, *NS* non-spirituality									

In the present study, this observation was confirmed by the strong correlation (*ρ* = 0.87;* p* < 0.001) between religiosity and spirituality in the second CFA performed. There is also the possibility that a partial overlap was obtained between both constructs due to 60.2% of the sample being made up of religious individuals (who tend to indicate low levels of NR and NS) and atheists (who tend to have high levels of NR and NS). The strong correlation between religiosity and spirituality leads us to some possible conclusions. One hypothesis is that, for Brazilians, unlike Americans, the concepts of religiosity and spirituality are similar and closely related, which would explain the robust correlation observed between these variables. This contrasts with the original sample, which found a correlation of lower intensity (0.64). However, the results seem to reflect a plausible cultural reality, if we take into account that the USA and Europe have cultural contexts in which spirituality is more naturally detached from religion (Keysar, 2017). Furthermore, it is important to highlight that we did not find studies, with a sufficiently representative sample, conducted in Brazil that confirm this similarity between the concepts, suggesting a promising direction for future research on this topic.

The Brazilian NRNSS exhibited a favorable validity based on the relationships with external measurements. The emphasis on the strong and negative correlation between intrinsic religiosity and non-religiosity (*ρ* = − 0.885; *p* < 0.01) may indicate that the items that constitute the non-religiosity factor are formulated in such a way that they report more on the dimension of the central experience of religion in the lives of individuals (Allport & Ross, 1967). This is corroborated by the similarity in the correlation between non-spirituality and intrinsic religiosity (*ρ* = − 0.864; *p* < 0.01). As in the CFA results, such data point to a possible connection with Pargament’s conceptual perspective of spirituality (Zinnbauer & Pargament, 2005), seen as a genuine experience of religiosity, given the fact that a greater degree of intrinsic religiosity is related to lower levels of non-religiosity and non-spirituality.

Satisfactory results were obtained for validity based on the external relations with the SRPB scale. As shown by Fleck and Skevington (2007), an important nuance of the instrument is the fact that it does not directly measure the construct of spirituality, but rather some of its central components. Our results showed strong negative correlations between NRNSS factors and those that are central and exclusive aspects of spirituality, as they have implicit or explicit assumptions of spiritual dimensions in their formulations (i.e., connection with spiritual being, spiritual strength, and faith). There was also a negative and strong significant correlation between the NR and NS factors and the meaning of life. Although some studies have presented data that reinforce the evidence of a lower level of meaning in life in nonreligious individuals (Abeyta & Routledge, 2018; Nelson et al., 2021), there is no consensus on this (Speed et al., 2018; Uzarevic & Coleman, 2021). Even so, a similar correlation was observed when this scale was translated into Greek (Polemikou et al., 2019).

The one-way ANOVA provided important data for the justification and validity of the NRNSS. The results reinforced the validity of the NRNSS, indicating a decreasing order in the level of non-religiosity and non-spirituality as people confirmed that they were, respectively, atheist, agnostic, spiritual but not religious, and religious. We also found that the population with the greatest distinction between the levels of non-religiosity and non-spirituality were spiritual but not religious people, presenting a relatively high level of non-religiosity (*M* = 3.92; SD = 0.69) and low level of non-spirituality (*M* = 2.40; SD = 0.82; see Table 5). This finding provides additional evidence of validity from a conceptual perspective. This result corroborates the comparisons between religious and spiritualists, where the first ones obtained an expressively higher degree of NR and a subtle difference in the NS degree (see Table 6).

Our study provides two important insights. First, despite spirituality being strongly linked to religiosity, as argued by Zinnbauer and Pargament (2005), the measurement of both separately can be extremely fruitful, taking into account that there is a population that scores high in one, but not in the other construct. Second, the NRNSS is sensitive enough to capture those who identify themselves as atheists, agnostics, spiritual but not religious, and religious.

Despite the existence of some R/S instruments for the Brazilian context (Forti et al., 2020), this is the first one that seeks to separately investigate (non)religiosity and (non)spirituality in a single questionnaire. This instrument arises from the need for scales configured to identify and investigate the nonreligious population, without the items being built on implicit assumptions about the participants or paradoxical statements that make it difficult for participants to answer, potentially adding bias to participants’ responses (Cragun et al., 2015; Meezenbroek et al., 2012; Zwingmann et al., 2011).

Our study has some limitations, including the small number of spiritual but not religious (SBNR) in the sample. The small number of SBNR individuals contrasts with the large number of atheist and religious respondents and may have influenced the results to show a higher correlation between religiosity and spirituality than may be observed when looking at just spiritualist individuals. Another limitation is that a significant portion of the sample was composed of people with high levels of education, which indicates our data are not representative of the Brazilian population generally. Yet another sampling limitation is that we used a non-probabilistic convenience sample. Despite the benefits of this sampling method, this decision may compromise the representativeness of the data, limiting the generalizability of the results to the Brazilian population as a whole. Although the study provides valid data for the new scale in the Brazilian context, it is recommended that future research use representative samples to corroborate and expand the conclusions presented in this study, ensuring greater validity and reliability.

Finally, a last limitation to consider is that the item regarding religious/spiritual identity was administered before the main scale. This sequence may have influenced the responses to some extent due to context effects, potentially inflating the internal consistency of the scale and impacting the results of the analyses. Future studies should consider placing the religiosity question after the scale.

## Conclusions

The Brazilian NRNSS presented favorable psychometric properties, showing the possibility of its application in different contexts. Additionally, the NRNSS is not limited to the investigation of the nonreligious population; it can provide useful insights when studying those who consider themselves religious. It is important to emphasize that the NRNSS, in addition to the objective of identifying the nonreligious population, seeks to cover the spectrum that varies from the presence to the absence of religiosity and spirituality.

We propose that more research be carried out with the Brazilian NRNSS using representative samples. This would provide additional insights into the levels of religiosity and spirituality in Brazil, generally. We also propose that in future studies that seek to compare the results of the NRNSS between the groups investigated, an item should be used to understand why the participant indicates that they do not identify with any of the (non-)religious identities, as this could generate insights into possible weaknesses in the measures. Additionally, representative samples may illustrate whether level of education is important for understanding the NRNSS and whether the factors measured by the NRNSS vary by level of education. Finally, it is believed that Brazilian NRNSS has the potential to offer new possibilities for research on religion, spirituality, and health in Brazil, separately identifying religiosity and spirituality. Having clear, valid, and reliable measures of these constructs may prove fruitful in studying physical, social, and mental health.

## Data Availability

The datasets used and/or analyzed during the current study are available from the corresponding author on reasonable request.

## Abbreviations

ANOVA: Analysis of variance
CFA: Confirmatory factor analysis
CFI: Comparative Fit Index
EFA: Exploratory factor analysis
NR: Non-religiosity
NS: Non-spirituality
NRNSS: Non-religious non-spiritual scale
RDWLS: Robust diagonally weighted least squares
RMSEA: Root mean square error of approximation
SRMR: Standardized root mean residual
TLI: Tucker-Lewis Index
